# Thoughts Falling Apart: Disorganized Schizotypy Specifically Predicts Both Psychotic‐ and Stress‐Reactivity in Daily Life

**DOI:** 10.1111/jopy.13019

**Published:** 2025-03-11

**Authors:** Levente Rónai, Flóra Hann, Szabolcs Kéri, Bertalan Polner

**Affiliations:** ^1^ Institute of Psychology, ELTE Eötvös Loránd University Budapest Hungary; ^2^ Institute of Psychology University of Szeged Szeged Hungary; ^3^ Sztárai Institute University of Tokaj Sárospatak Hungary; ^4^ Department of Physiology Albert Szent‐Györgyi Medical School, University of Szeged Szeged Hungary; ^5^ Donders Institute for Brain, Cognition and Behaviour Radboud University Nijmegen the Netherlands

**Keywords:** experience sampling method, psychotic‐like experiences, schizotypy, stress‐reactivity

## Abstract

**Objective:**

Schizotypal personality traits, such as unusual experiences, odd beliefs, or social anhedonia, predict psychotic‐like experiences (PLEs) and heightened stress‐reactivity in daily life. Yet, in previous studies, stressor appraisal, but not *exposure*, was used to predict stress‐reactivity, which might be a consequence of behavioral sensitization rather than a valid predictor of it.

**Method:**

We conducted an experience sampling study where 126 participants reported PLEs, event appraisals, and exposure to stressors, yielding 4611 observations. We tested the association of schizotypal traits with PLEs and event‐unpleasantness in interaction with stressor exposure.

**Results:**

Disorganized (but not positive or negative) schizotypy predicted not only more intense PLEs but also higher PLEs in periods when stressor exposure had risen. However, in higher negative schizotypy, such PLE‐reactivity to stressors was reversed. Moreover, individuals with higher disorganization found events more unpleasant overall, and for them, being exposed to more stressors was related to a steeper rise in appraising events as unpleasant.

**Conclusions:**

Disorganization, but not positive or negative schizotypy, might be a specific determinant of stressor‐related increases in PLEs and negative event appraisal in everyday life in the general population. This supports that disorganized personality might be a critical predictor of vulnerability to stress‐related mental health impairments.

## 1 Introduction

Reality distortions are more common than one might expect. Roughly one in 20 individuals in the population report experiences resembling psychosis during their lifetime (McGrath et al. 2015; van Os et al. 2008). Two in a hundred will report their first psychotic experience within a year, regardless of their age (Staines et al. 2023). Psychotic experiences and milder psychotic‐like experiences (PLEs) (Seiler et al. 2020) both predict future psychotic disorders (Lindgren et al. 2022; Linscott and van Os 2013). They are also associated with subsequent suicidal thoughts and behaviors (Bromet et al. 2017), or any mental disorder (Lindgren et al. 2022), and co‐occur with increased depression, anxiety, impoverished functioning, and reduced well‐being (Austin et al. 2023), and a history of virtually any mental disorder (Bourgin et al. 2020). Relatedly, individuals with psychotic experiences report using mental health services more often (Bhavsar et al. 2018), and PLEs are related to a stronger intention to seek treatment (Bridgwater et al. 2023). Thus, it seems plausible that PLEs could signal a non‐specific vulnerability for mental distress (Stochl et al. 2015), providing a broader motivation for characterizing their antecedents.

Psychosis and PLEs can be triggered by environmental stressors as a function of neurodevelopmental vulnerability (Howes and Shatalina 2022; Pruessner et al. 2017). Studies showed that psychosis vulnerability and stress‐reactivity are positively related (Collip et al. 2008; Myin‐Germeys and van Os 2007), and distress induced stronger psychotic experiences among individuals with psychotic disorder than healthy controls and unaffected relatives of the patients (Klippel et al. 2022). In line with this, individuals with a high risk of psychosis show increased distress‐induced psychotic reactivity (i.e., behavioral sensitization; Collip et al. 2008) compared with healthy controls (Klippel et al. 2017; Reininghaus et al. 2016). Additionally, a polygenic risk score for schizophrenia (PRS) predicted a stronger association between stress and psychotic experiences in healthy controls (Schick et al. 2022). However, in unaffected siblings of individuals diagnosed with schizophrenia, a higher PRS was related to reduced psychotic reactivity. Thus, it is unclear which are the most informative indicators of trait vulnerability to psychosis that predict the emergence of psychosis under stress. Such insight would be valuable for the prevention of psychotic disorders.

Schizotypy provides a framework to study the causal mechanisms leading to schizophrenia within a personality psychology approach (Barrantes‐Vidal et al. 2015). Schizotypy, conceptualized by Radó (1953) and refined by Meehl (1962, 1989), refers to a set of personality traits that resemble the signs and symptoms of schizophrenia. Importantly, schizotypy predicts future risk of schizophrenia‐spectrum disorders (Debbané et al. 2015) and shows similarities with schizophrenia in terms of cognition and brain structure and function, even in healthy individuals (Ettinger et al. 2012, 2014; Nelson et al. 2013; Siddi et al. 2017; Steffens et al. 2018). This provides a rationale for studying healthy individuals in order to characterize causal mechanisms that may lead to psychosis. Furthermore, the relevance of the schizotypy framework goes beyond schizophrenia risk in that it is well‐aligned not only with broader transdiagnostic dimensional models of psychopathology such as the Hierarchical Taxonomy of Psychopathology (HiTOP) (Cicero et al. 2022; Kotov et al. 2020; Ringwald et al. 2021) but also with models of normal personality variation as well (Blain et al. 2020; Chmielewski et al. 2014).

Schizotypy has multiple dimensions, which parallel the symptom dimensions of schizophrenia (Gross et al. 2018; Kotov et al. 2016, 2020; Kwapil, Gross, Silvia, et al. 2018). Tendencies to experience reality distortion in milder forms, such as unusual perceptions and weird beliefs, characterize *positive schizotypy*. Reduced motivation and pleasure capacity, associated with apathy and social withdrawal, comprise *negative schizotypy*. Finally, subtle impairments resembling thought disorder, such as difficulties controlling attention, thoughts, and behavior, make up *disorganized schizotypy*. Disorganization is of particular interest here: According to seminal theories, it is a core feature of the schizophrenia‐risk phenotype that is proximal to the neurodevelopmental risk factors (Cornblatt and Keilp 1994; Meehl 1989). This conjecture is supported by network studies showing that disorganization tends to be highly central (Brasso et al. 2023; Christensen et al. 2018; Polner, Faiola, et al. 2021).

There are different concepts related to the representation of schizotypal traits on the illness–health and mood–psychosis spectra (Grant et al. 2018), and it is still debated how schizotypal traits are distributed in the general population and whether at all they can be related to adaptive functioning (Barrantes‐Vidal and Kwapil 2023). It has been put forward that individuals high in positive schizotypy but low in negative and disorganized schizotypy show enhanced positive affect, creativity, and spirituality (McCreery and Claridge 2002; Mohr and Claridge 2015; Polner, Hupuczi, et al. 2021) although such individuals are not completely free of mental health problems, either.

Accordingly, previous studies pointed out that increased positive schizotypy predicts elevated PLEs (Kwapil et al. 2012, 2020; Kemp et al. 2024) and greater stress‐related increases in PLEs (PLE‐reactivity) (Barrantes‐Vidal et al. 2013) in daily life. However, other studies drew attention to disorganized schizotypy: It specifically predicts increased negative and decreased positive affect (Kwapil et al. 2020) and increased variability, instability, and reactivity of negative affect (Kemp et al. 2023). Curiously, in both of these studies, when disorganized schizotypy was not accounted for, elevated and more variable negative affect was predicted by positive schizotypy (Kemp et al. 2023; Kwapil et al. 2020). In addition, disorganized schizotypy predicted both positive and disorganized elements of daily life PLEs, while positive schizotypy only predicted the positive domain of PLEs in everyday life (Kwapil et al. 2020). Moreover, disorganized schizotypy predicted psychotic‐like experiences (PLEs), rumination, sleep disruption, and somatic complaints during the COVID pandemic (Simor et al. 2021). In another study, general and disorganized schizotypy predicted increased negative emotionality of dreams, while positive schizotypy was linked to more salient dreams (Báthori et al. 2022). Relatedly, disorganization predicted increased PLE‐reactivity induced by a standardized lab‐based social stress paradigm (Grant and Hennig 2020). Recently, Kemp et al. (2024) also reported that disorganized schizotypy uniquely predicted PLE‐reactivity in daily life, while positive schizotypy was related to mean PLE intensity. In their robust study, they recruited a large, heterogeneous sample and analyzed subdimensions of PLEs as well. At the same time, they measured stressful and positive situation *appraisals* with single items that did not specify the type of stressors and were completed together with the items assessing PLEs. Therefore, the literature lacks a high‐resolution measurement of the *exposure* to specific social, economic, and health‐related stressors as well as an analysis of the latter's relationship with PLEs as a function of schizotypal traits.

More specifically, we argue that there might be a hidden flaw in the operationalization of PLE‐ and stress‐reactivity. Instead of reporting stressor exposure, participants often appraise recent significant events, and then researchers evaluate how strongly these appraisals predict (subsequent) negative affect and PLEs (see Kemp et al. 2024; Myin‐Germeys et al. 2001; Myin‐Germeys and van Os 2007; Reininghaus et al. 2016). However, it is plausible that such subjective evaluations are rather consequences of behavioral sensitization and not independent predictors of it. Thus, differentiating between reactivity to exposure to stressful events vs. their subjective evaluation seems justified from the perspective of construct and predictive validity, and their associations with schizotypal traits remain to be tested.

### 1.1 Aims and Hypotheses

Positive schizotypy is a robust predictor of PLEs in daily life (Kwapil et al. 2012, 2020; Kemp et al. 2024). However, disorganization is a central trait of psychosis vulnerability (Brasso et al. 2023; Christensen et al. 2018; Cornblatt and Keilp 1994; Meehl 1989; Polner, Faiola, et al. 2021) that predicts PLE‐reactivity in the laboratory (Grant and Hennig 2020) and in daily life (Kemp et al. 2024). Furthermore, previous studies on schizotypal traits and PLE‐reactivity operationalized the latter by regressing PLEs on subjective appraisals without considering stressor exposure (Barrantes‐Vidal et al. 2013; Kemp et al. 2024). Here, we build on these seminal studies and address these outstanding questions. Aligned with previous studies (Kwapil et al. 2012, 2020; Kemp et al. 2024), we hypothesize that positive schizotypy per se will predict PLEs, event appraisal, and PLE‐ and stress‐reactivity in daily life. However, we assume that when accounting for it, disorganization will be the strongest predictor of PLE‐ and stress‐reactivity in real life. This expectation is formulated based on findings emphasizing the centrality and predictive value of disorganization in psychosis vulnerability (Brasso et al. 2023; Christensen et al. 2018; Grant and Hennig 2020; Polner, Faiola, et al. 2021). Given previous findings (Kemp et al. 2023), we expect no association of negative schizotypy either with mean levels of PLEs and unpleasantness ratings or with PLE‐ and stress‐reactivity. If our hypothesis is confirmed, it will establish that disorganized schizotypy is a distinguished indicator of underlying vulnerability to schizophrenia spectrum disorders.

## 2 Methods

### 2.1 Participants

**FIGURE 1:**
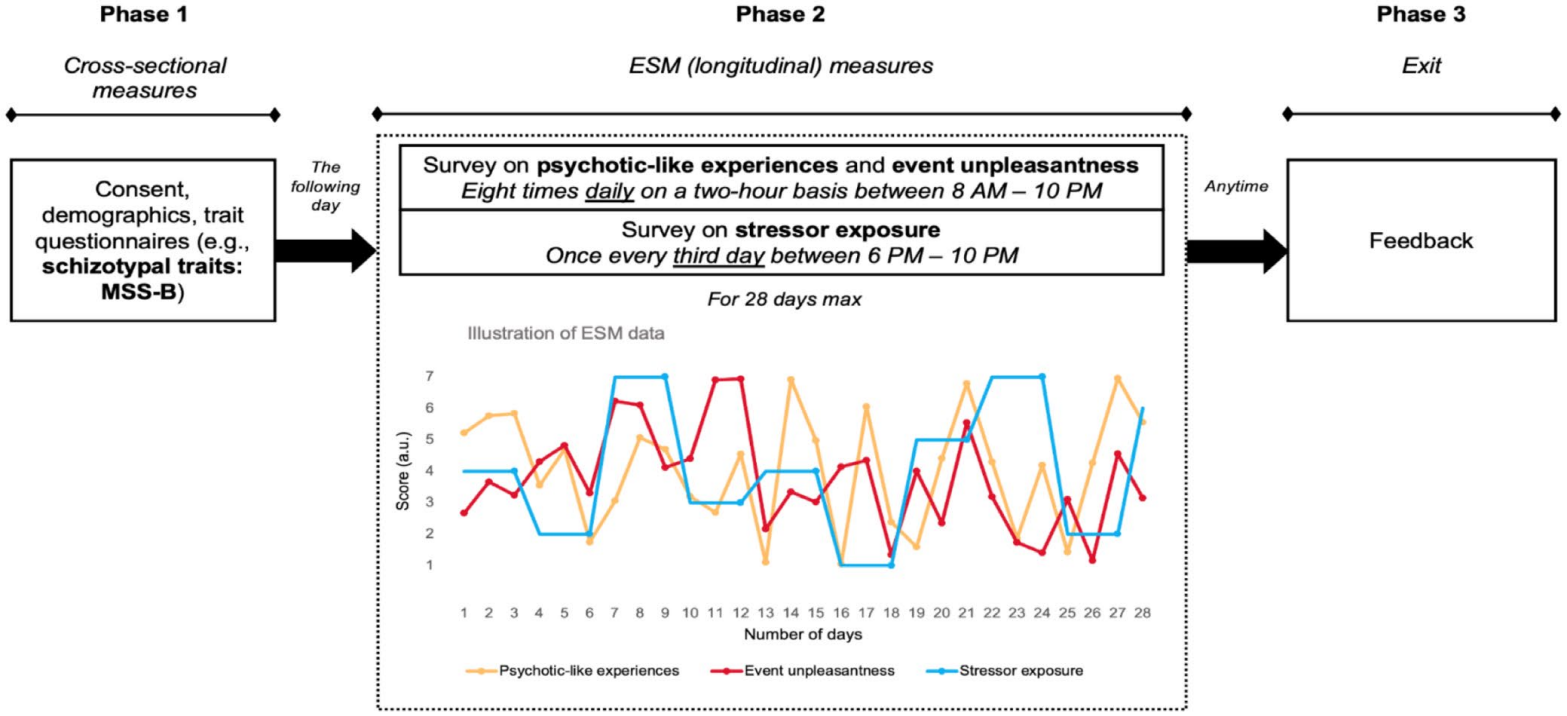
The design of the study. After completing the cross‐sectional assessment (Phase 1), participants could decide to enter the longitudinal section (Phase 2), which started the next morning. This section comprised 8 prompts administered on a set schedule every two hours daily for short surveys on psychotic‐like experiences and event‐unpleasantness (yellow and red lines indicate the means of daily measurements). Every third day, a 5‐min survey was sent to evaluate stressor exposure in the past 3 days (blue line). In the figure, dots indicate occasions when constructs were assessed. Participants could proceed for up to 28 days but could quit at any point (Phase 3). Upon request, they received feedback containing figures that showed the daily fluctuation of their sleep quality and quantity, and their affective states (these constructs were also assessed in the ESM surveys, but are not analyzed in the present study).

Our sample comprised Hungarian‐speaking individuals from the general population. They were recruited through press releases and social media posts during late Spring and early Summer of 2021. First, 221 participants completed a cross‐sectional baseline assessment (N[female] = 163, mean[age] = 40.44 years, SD[age] = 14.24 years, min[age] = 18, max[age] = 78). One hundred and eighty‐one participants from this initial pool proceeded to the longitudinal phase of the study. This second, ESM phase involved short surveys that were sent on a set schedule every two hours eight times a day, and a slightly longer (5‐min) survey once every third day (see more information on the study design in Figure 1 and the Design section). The total number of surveys administered to each participant varied, as they could quit the study at any time. See the Results section for information on compliance rates.

We retained participants who completed at least one short daily survey and a longer survey in the same three‐day period, as our key analyses required data from both types of surveys. Furthermore, individuals responding too fast were excluded to ensure data quality (see “Quality control at baseline” section in the Supporting Informations). The final sample comprised 126 participants, whose demographic characteristics are reported in Table 1 (except a small, but significant difference in age, there were no significant differences in comparison to participants not included, see Tables S1 and S5). They completed a total of 4611 ESM surveys (per capita: Median = 30, Obs. Range = 1–176, Theor. Range = 0–224).

**TABLE 1:** Demographic characteristics of the sample.

Sample descriptives	
Size	126
Age	
Mean	42.49
Median	44
SD	15.43
Range	18–78
Sex	
Female % (*N*)	77% (97)
Male % (*N*)	23% (29)
Education	
Primary school or lower % (*N*)	0.79% (1)
Vocational school without high school diploma % (*N*)	0% (0)
High school diploma or equivalent % (*N*)	24.6% (31)
Bachelor's or Master's degree % (*N*)	68.25% (86)
Doctorate (PhD) % (*N*)	5.56% (7)
Other % (*N*)	0.79% (1)

Since the study was designed to reach the general population, participation in the study was mainly advertised through social media platforms and the most visited online news portals in the country. Therefore, the sample size was constrained by the available resources, and based on the standards of the ESM literature, we aimed to enroll about 100–200 participants. Still, to ensure that we had the statistical power to detect the effects we aimed to test, we performed a post hoc statistical calculation using the R package “PowerAnalysisIL” (Lafit et al. 2021) (v0.1.0). The analyses revealed that sufficient power was available to capture the main effects of disorganization and its interaction with stressor exposure in our models (in both cases, power = 100%) (see Supporting Informations for more details).

Participation was voluntary, and participants provided informed consent. The authors assert that all procedures contributing to this work comply with the ethical standards of the relevant national and institutional committees on human experimentation and with the Helsinki Declaration of 1975, as revised in 2008. The study was approved by the United Ethical Review Committee for Research in Psychology, Hungary (reference number: 2021‐38). This study was not preregistered.

### 2.2 Instruments and Design

#### 2.2.1 Cross‐Sectional Phase

The first, cross‐sectional phase of the study contained questionnaires assessing trait‐level constructs. Participants also provided demographic information and answered questions regarding their socioeconomic status, living arrangements, and exercise habits. In this phase, schizotypal personality traits were assessed with the Hungarian version of the Multidimensional Schizotypy Scale—Brief (MSS‐B) (Gross et al. 2018; Kwapil, Gross, Silvia, et al. 2018). The scale encompasses 38 self‐report “yes/no” items grouped into three subscales: (I) a *positive* dimension (13 items; paranoia, odd beliefs and perception), (II) a *negative* dimension (13 items; anergia, anhedonia and flattened affect), and (III) a *disorganization* dimension (12 items; disturbances in organization and expression of behavior and thoughts).

We tested the factor structure of MSS‐B with confirmatory factor analysis (CFA) using robust WLSMV estimation (“lavaan” R package (Rosseel 2012), v0.6‐13). Two items (*“If given the choice, I would much rather be with another person than alone”* [10th item, negative dim.] and *‘I often think that I hear people talking only to discover that there was no one there’* [5th item, positive dim.]) were excluded from the model due to issues related to negative variances. Overall, the model had convincing fit indices; however, SRMR appeared to be high (*χ* ^2^(591) = 750.310, *p* < 0.001, CFI = 0.975, TLI = 0.973, RMSEA = 0.039, SRMR = 0.178). Ordinal Cronbach's alphas (Zumbo and Kroc 2019) indicated that the subscales of MSS‐B had convincing reliability in the sample (ordinal alpha[positive dim.] = 0.78, ordinal alpha[negative dim.] = 0.88, ordinal alpha[disorg. dim.] = 0.96). The computed factor scores of MSS‐B subscales were used in further analyses (see Tables S3 and S4 in Supporting Information for details).

#### 2.2.2 ESM Phase

##### 2.2.2.1 Psychotic‐Like Experiences

In the ESM surveys sent every two hours, participants repeatedly evaluated their psychotic‐like experiences in the preceding two hours on a 7‐point Likert scale ranging from “not at all” to “completely.” Following previous studies (Cristóbal‐Narváez et al. 2017), we included eight items regarding *feeling suspicious, feeling mistreated, unusual senses, unusual thoughts, losing control, thoughts controlled by others, familiar things seeming strange* and *hearing/seeing things others could not*. Due to an error, two items that were used in previous studies (*feeling weird* and *hearing or seeing things others could not*) were left out from the ESM surveys. To examine the reliability of the PLE scale, we conducted a multilevel CFA with “lavaan” R package (Rosseel 2012) (v0.6‐13). The scale showed excellent psychometric properties in the sample (*χ* ^2^(37) = 890.054, *p* < 0.001, CFI = 0.935, TLI = 0.9, RMSEA = 0.055, SRMR[within‐person] = 0.041, SRMR[between‐person] = 0.077). The reliability of this scale was convincing both within (*ω* = 0.76) and between (*ω* = 0.86) individuals. The multilevel factor scores (i.e., the sum of between and within factor scores for each observation by participants) of PLE obtained in this model were used for statistical modeling (see Table S5 in Supporting Informations for details).

##### 2.2.2.2 Event‐Unpleasantness

In the ESM phase, participants also rated the pleasantness of the most significant event in the past two hours on a 7‐point Likert scale, 1 being “not at all pleasant” and 7 indicating “entirely pleasant.” Values of this item were reversed to capture subjective appraisal of adverse environmental stress (event‐unpleasantness).

##### 2.2.2.3 Exposure to Stressors

We measured exposure to social, economic and health‐related stressors in the survey sent out on a three‐day basis. The 20‐item questionnaire contained examples of events and situations that objectively evoke distress (e.g., losing one's job, conflict at work, health issues, financial difficulties) or provide a source of support (e.g., new job, improvement in health or finances; for details, see ‘Measurement of stressor exposure’ in Supporting Informations). The indicator extracted from this instrument was the mean of the responses to the items. To capture the effect of change in exposure to stressors (as opposed to differences in reporting stressor exposure on average), the obtained scores were centered within individuals.

The validity of this scale was established by testing whether it shows the expected positive associations with theoretically relevant criterion variables: PLEs and event‐unpleasantness. We fitted two baseline models (that were detrended and controlled for age and sex), where the predictor was the mean score of the items of the stressor scale (within‐individual‐centered). The dependent variable was daily life PLEs or event‐unpleasantness (see Results). Higher scores on the stressor scale significantly predicted both higher PLEs and event‐unpleasantness within individuals, confirming the predictive validity of the scale. We bootstrapped these models and found the predictive value of the mean score of the stressor scale was stable for both criterion variables (see Results). One may argue that the stressor items do not tap into manifestations of a latent variable but capture presumably independent events. In other words, separate stressors (e.g., the sickness of a loved one and a workplace conflict) do not stem from a common cause; hence, they are not expected to co‐occur. Thus, we deemed it would not be meaningful to analyze the internal structure or the reliability of the scale with latent variable models or indicators of internal consistency reliability.

#### 2.2.3 Design

We ran the study in the browser‐based application formr.org (Arslan et al. 2020), used for collecting time‐series data through an ESM design. On entering the study, participants completed a battery of trait questionnaires and provided demographic information. At the end of this section, participants could decide to enroll in the longitudinal phase of the study, which started the next morning. Prompts were sent out via email 8 times a day, on a set schedule, every two hours on the hour between 8:00 AM and 10:00 PM. These short surveys included the previously described items on psychotic‐like experiences and event‐unpleasantness in the preceding two hours, as well as other items not analyzed here (e.g., sleep quality and quantity). After the survey, participants completed two short cognitive tasks, approximately 1.5 min each, in randomized order. These tasks are not included in the present analysis.

Every third day, participants completed a 5‐min survey assessing depressive symptoms, support, and stressor exposure in the past 3 days (only the latter was analyzed here). This survey was sent at 6:00 PM and could be completed until 10:00 PM. On these days, participants received no further prompts; the next email was sent at 8:00 the following morning.

Participants could stay in the study for up to 28 days but could quit at any point. After participating for a minimum of 7 days, they could opt for feedback containing figures about the daily fluctuation of their negative and positive affective states, as well as their sleep quality and quantity. Note that this served as an incentive and did not mean a requirement to participate for a minimum of 7 days. The design of the study is depicted in Figure 1.

### 2.3 Statistical Analyses

Analyses were performed with R (v4.2.1; 2022) in RStudio (v2022.07.1; 2022). Data and code are openly available here: https://osf.io/umn5v/. We fitted linear mixed‐effects models using the R package lme4 (Bates et al. 2015) (v1.1‐31) to test the associations between schizotypal traits and daily life stress as well as PLE‐reactivity within and between individuals.

To test our hypotheses, we fit two sets of models with the same predictors but two different outcome variables: one set for PLEs and another set for event‐unpleasantness. We built up the sets using the following strategy: first, as baseline models, daily life PLEs/event‐unpleasantness were the outcome variables, and three‐day social and economic stressor exposure was the predictor variable. Their statistical relationships represent daily life PLE‐reactivity and stress‐reactivity, respectively. Second, we extended this model with negative and positive schizotypy and their interaction with stressor exposure. Note that in these models, we investigated the associations between schizotypal traits and stress‐ and PLE‐reactivity without accounting for disorganized schizotypy. Finally, disorganized schizotypy and its interaction with stressor exposure were also included as predictors of daily life PLEs and event‐unpleasantness.

We analyzed 4611 observations from 126 participants in the final models. All models were adjusted for age and sex, as they are associated both with negative emotionality (Donnellan and Lucas 2008; Weisberg et al. 2011) and schizotypy (Mason and Claridge 2006). The models were also detrended for the number of days that passed since entering the study and the time points within days (daily ESM assessment sessions, referred as “beeps”). In order to capture the effects of within‐person fluctuations, stressor exposure was within‐person centered (Hamaker and Grasman 2015). The models were fitted with random intercepts for each participant and random slopes for stressor exposure.

Assumptions of linearity, homogeneity of variance, normal distribution of residuals, random effects, and multicollinearity were tested. Assumptions were met in the stress‐reactivity models. However, non‐normality of residuals and heteroscedasticity were observed in the PLE‐reactivity models. Therefore, to test the stability of all models, we used “Wild” bootstrapping (with 5000 iterations, using the lmeresempler R package (Loy et al. 2023) [v0.2.4]), since it has been suggested to be sensitive to heteroscedasticity and non‐normality of residuals (Arghyrou and Gregoriou 2007; Flachaire 2005). The bootstrapping analysis confirmed the findings (for further details see Results). We visualized the bootstrapped distributions (Figure 4); note that these can be interpreted as an approximation of a noninformative nonparametric posterior probability distribution (Hastie et al. 2009). To assess the relative strength of the association positive vs. disorganized, as well as negative vs. disorganized schizotypal traits have with the outcomes of interest, we constructed bootstrapped distributions of the differences of the coefficients of their main effects and interactions with stressor exposure too (for more details, see Figure 4).

## 3 Results

### 3.1 Compliance and Descriptive Statistics

The median compliance rate for the short two‐hour daily surveys in the final sample of 126 participants was 41.7%^1^ (Mean = 39.3%, Min = 0.5%, Max = 78.2%, SD = 18.8%, see Figure S1 in the Supporting Informations). The median compliance on the three‐day surveys was 80% (Mean = 75.9%, Min = 11.1%, Max = 100%, SD = 21.7%, see Figure S2 in the Supporting Informations). The median number of days spent in the ESM phase was 21 (Mean = 17.7 days, Min = 2 days, Max = 27 days, SD = 9.3 days). We found no significant correlations between compliance rates for either survey type and MSS‐B subscales (see results in Table S2).

**FIGURE 2:**
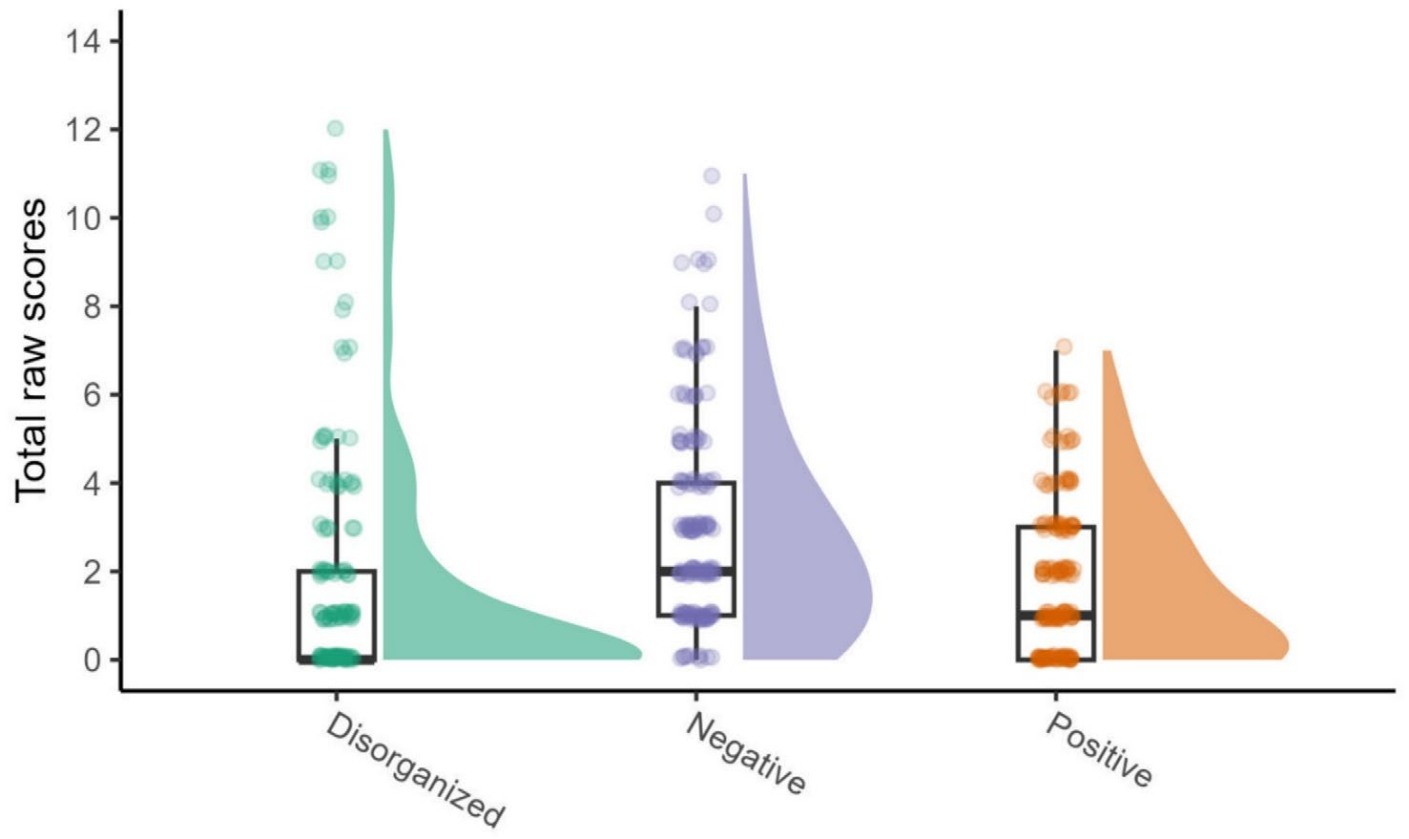
Distributions of raw sum scores of the MSS‐B subscales in the final sample.

Descriptive statistics of the final sample are shown in Table 2 and Figure 2 (see further details in the Supporting Informations, Figure S3, and Table S6). We observed a wide range of variability on all schizotypal dimensions that were all measured reliably in the sample (see Methods), and the descriptives were comparable to other studies using the MSS‐B (Gross et al. 2018) or the full MSS (Sahakyan et al. 2021).

**TABLE 2:** Descriptive statistics.

	Mean	SD	Obs. range	Theor. range
Psychotic‐like experiences^a^	9.76	4.44	8 to 40	8 to 56
Event‐unpleasantness	3.23	1.522	1 to 7	1 to 7
MSS‐B subscales				
Disorganized	1.74	2.86	0 to 12	0 to 12
Positive	1.54	1.82	0 to 7	0 to 13
Negative	2.71	2.52	0 to 11	0 to 13
Stressor exposure^b^	0.02	0.2	−0.6 to 0.9	−1 to 1

^a^ Raw sum scores of psychotic‐like experiences are displayed. Note that scores based on multilevel confirmatory factor analyses were used for modeling.

^b^ Raw mean scores of the stressor exposure scale items are displayed. Note that positive scores represent higher distress, while negative mean scores on stressor items indicate improvements in health‐related, financial, and social conditions.

### 3.2 Associations Between Schizotypy Dimensions and PLE‐Reactivity

#### 3.2.1 Establishing Daily Life PLE‐Reactivity Within Individuals

First, we focused on the effect of stressor exposure on PLEs, that is, PLE‐reactivity. In the baseline model, we demonstrated PLE‐reactivity: higher stressor exposure predicted higher PLEs within individuals, and this result was further supported by the bootstrapping analysis (Standardized Beta = 0.06, *p* < 0.05, Bootstrapped Raw Est. [95% CI] = 0.63 [0.002; 1.26]). Earlier time of day (i.e., a smaller beep) (Standardized Beta = −0.02, *p* < 0.05, Bootstrapped Raw Est. [95% CI] = −0.02 [−0.03; −0.002]) and more days spent in the ESM phase (Standardized Beta = 0.02, *p* < 0.05, Bootstrapped Raw Est. [95% CI] = −0.004 [−0.007; 0.015]) also predicted higher PLEs.

#### 3.2.2 Positive but Not Negative Schizotypy Predicts Daily Life PLE‐Reactivity Within Individuals

Then, we extended the above model with negative and positive schizotypal traits and their interactions with stressor exposure (Table 3). Study beep's main effect on PLEs remained significant. In addition, higher trait‐level positive schizotypy predicted higher daily life PLEs. Positive schizotypy did not moderate PLE‐reactivity, as indicated by the nonsignificant interaction. Negative schizotypy did not predict either PLEs or PLE‐reactivity.

**TABLE 3:** Results of multilevel models where schizotypal traits predict momentary PLEs/event‐unpleasantness (main effects) and PLE‐reactivity/stress‐reactivity (interactions). See also Figure 2.

Predictors	Dependent variables			
	PLEs	PLEs	Event‐unpleasantness	Event‐unpleasantness
	Bootstrapped Est [95% CI]	Bootstrapped Est. [95% CI]	Bootstrapped Est. [95% CI]	Bootstrapped Est. [95% CI]
Day	0.004 [−0.007; 0.014]	0.004 [−0.007; 0.014]	**−0.008 [−0.017; 0.002]** **	**−0.008 [−0.017; 0.002]** **
Beep	**−0.013 [−0.025; −0.001]** *	**−0.013 [−0.02; −0.002]** *	**−0.034 [−0.058; −0.011]** ***	**−0.035 [−0.058; −0.012]** ***
Gender [Male]	−0.199 [−0.478; 0.066]	−0.177 [−0.438; 0.076]	0.005 [−0.429; 0.426]	0.018 [−0.403; 0.428]
Age	−0.002 [−0.011; 0.008]	0.000 [−0.009; 0.009]	**−0.013 [−0.024; −0.002]** *	**−0.011 [−0.022; 0.000]** *
Stressor exposure	0.579 [−0.003; 1.142]	0.375 [−0.113; 0.822]	**0.616 [0.242; 0.989]** **	**0.504 [0.159; 0.850]** *
Negative Sch.	−0.008 [−0.218; 0.201]	−0.183 [−0.39 7;0.031]	**0.383 [0.148; 0.617]** ***	0.244 [0.002; 0.490]
Positive Sch.	**0.480 [0.121; 0.834]** ***	0.236 [−0.103; 0.585]	0.000 [−0.225; 0.224]	−0.197 [−0.467; 0.077]
Stressor exposure × Neg. Sch.	−0.287 [−0.879; 0.284]	**−0.97 [−2.01; 0.02]** *	−0.171 [−0.656; 0.334]	−0.469 [−0.931; 0.000]
Stressor exposure × Pos. Sch.	0.567 [−0.233; 1.383]	−0.367 [−1.178; 0.430]	**0.559 [0.041; 1.066]** *	0.062 [−0.524; 0.641]
Disorganized Sch.		**0.439 [0.196; 0.677]** **		**0.356 [0.099; 0.607]** *
Stressor exposure × Disorg. Sch.		**1.849 [0.201; 3.552]** ***		**0.929 [0.194; 1.661]** *
*N*	126			
Observations	4611			
Marginal *R* ^2^/Conditional *R* ^2^	0.087/0.635	0.116/0.631	0.08/0.451	0.095/0.451

*Note:* Significant effects are highlighted in bold.

* *p* < 0.05.

** *p* < 0.01.

*** *p* < 0.001.

#### 3.2.3 Disorganized Schizotypy Is a Specific Predictor of Daily Life PLE‐Reactivity Within Individuals

**FIGURE 3:**
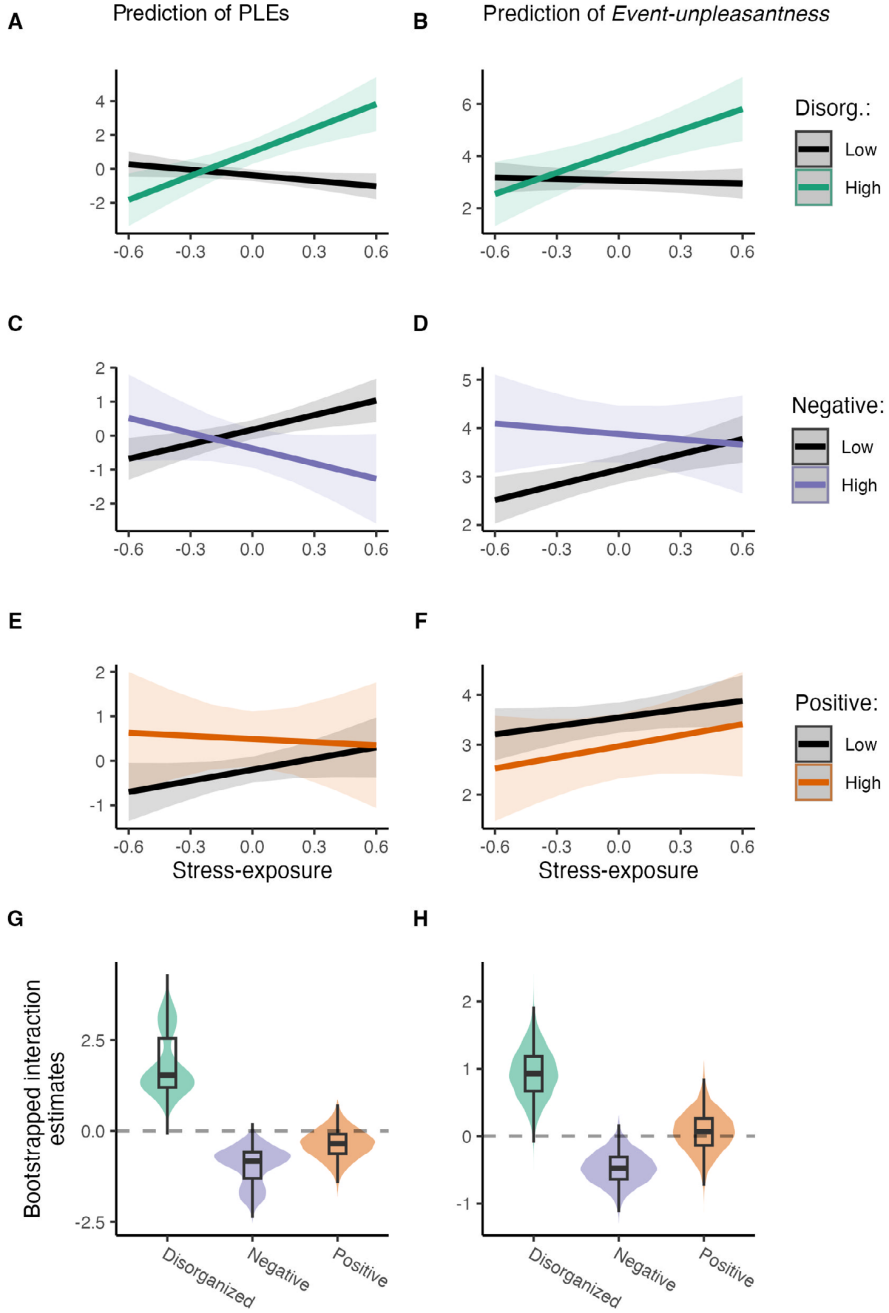
Interaction effects between schizotypal traits and stressor exposure on daily life PLEs (A, C, E) and event‐unpleasantness (B, D, F). Disorganized schizotypy significantly moderated PLE‐ and stress‐reactivity: Individuals with higher disorganized schizotypy reported higher levels of PLEs in association with exposure to distress (A) as well as they evaluated exposure to distress as subjectively more unpleasant (B). In order to test the stability of these interaction effects, we used ‘Wild’ bootstrapping (see G and H for the distributions and box plots of bootstrapped raw estimates) that confirmed our findings: Disorganized schizotypy's interaction effects with stressor exposure were in the positive range according to the 95% CIs of distributions (for CIs of bootstrapped raw estimates of stressor‐exposure—disorganized schizotypy interactions, see also Table 3).

Next, to test whether disorganized schizotypy predicts PLE‐reactivity in daily life above the effect of positive and negative schizotypal traits, we extended the previous model with disorganized schizotypy and its interaction with stressor exposure. Our analysis showed that higher disorganized schizotypal traits were associated with higher daily life PLEs (main effect) and PLE‐reactivity (interaction with stressor exposure). The effects of positive schizotypy did not remain significant after the inclusion of disorganized schizotypy. The effect of time of day (beep) on PLEs remained significant, whereas negative schizotypy significantly moderated the association between stressors and daily life PLEs: decreased negative schizotypy appeared to be related to higher levels of PLE‐reactivity. The moderation of PLE‐reactivity by schizotypal traits and the distributions of bootstrapped interaction coefficients can be seen in Table 3 and Figure 3 (first plot column: A, C, E, G).

**FIGURE 4:**
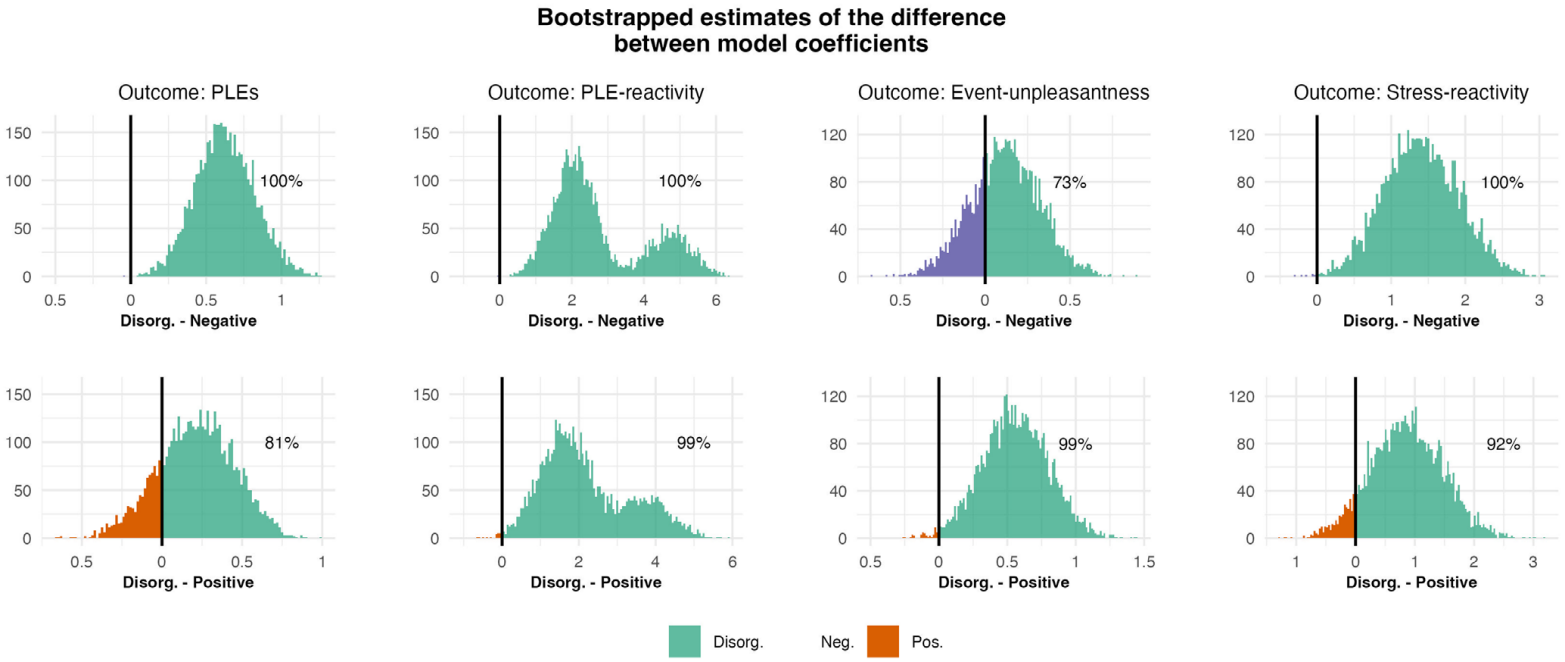
Distributions of differences between bootstrapped multilevel model coefficients (schizotypal traits' main and interaction effects on daily life PLEs, event‐unpleasantness, stress‐ and PLE‐reactivity). Differences in bootstrapped coefficient estimates (‘Wild’ bootstrapping, *N* [iterations] = 5000) for the effects of schizotypal traits (disorganization's coefficient minus the positive or negative trait's coefficient) on the outcome variables, which are indicated above each column in the figure. Percentages of bootstrap samples in which disorganization had a higher coefficient for the given outcome (as compared to negative or positive schizotypal traits) are shown next to the histograms. In each panel, the *X*‐axis shows the magnitude of these differences. The colors vary according to which schizotypal trait had a higher coefficient in predicting the outcome variable. Disorg., disorganization; Neg., negative schizotypy; Pos., positive schizotypy.

To assess the relative strength of the association of disorganized versus positive as well as disorganized versus negative schizotypal traits with PLEs and PLE‐reactivity, we also constructed bootstrapped distributions of the differences of the coefficients (Figure 4). Our results showed that, compared to the negative factor, disorganization was a stronger positive predictor of daily life PLEs and PLE‐reactivity in nearly 100% of the comparisons of the single bootstrapped estimates. Furthermore, compared with the positive factor, disorganization was found to be a stronger positive predictor of daily life PLEs in 81% of the differences in the bootstrapped coefficients. Moreover, for PLE‐reactivity, disorganization was found to be a stronger positive predictor in 99% of cases compared to the positive trait. However, *not only the proportion* of stronger positive estimates but also *the distributions* of the coefficient differences are relevant in this comparison: These are shown in detail in Figure 4.

### 3.3 Associations Between Schizotypy Dimensions and Stress‐Reactivity

#### 3.3.1 Establishing Stress‐Reactivity Within Individuals

Then, we turned our attention to the effect of stressor exposure on eventunpleasantness, that is, stress‐reactivity. First, we fitted a baseline model in which we demonstrated stress‐reactivity: higher stressor exposure significantly predicted higher event‐unpleasantness within individuals (Standardized Beta = 0.06, *p* < 0.001, Bootstrapped Raw Est. [95% CI] = 0.72 [0.29; 1.16]). Earlier time of day (i.e., a smaller beep) (Standardized Beta = −0.05, *p* < 0.001, Bootstrapped Raw Est. [95% CI] = −0.035 [−0.06; −0.01]) and fewer days spent in the ESM phase (Standardized Beta = −0.04, *p* < 0.01, Bootstrapped Raw Est. [95% CI] = −0.01 [−0.02; 0.002]) also predicted higher event unpleasantness. Moreover, higher age predicted lower daily life event‐unpleasantness within individuals (Standardized Beta = −0.16, *p* < 0.01, Bootstrapped Raw Est. [95% CI] = −0.016 [−0.03; −0.005]).

#### 3.3.2 Negative Schizotypy Predicts Daily Life Event‐Unpleasantness While Positive Schizotypy Is Associated With Higher Stress‐Reactivity Within Individuals

Then, we added positive and negative schizotypal traits and their interactions with stressor exposure (Table 3). Study days', beep's, age's, and stressor exposure's main effect on event‐unpleasantness remained significant. Furthermore, higher trait‐level negative schizotypy predicted higher daily life event‐unpleasantness. Positive schizotypy's main effect on event‐unpleasantness was not significant, while positive schizotypy moderated stress‐reactivity: Higher positive schizotypy predicted a stronger positive association between exposure to stressors and daily life event‐unpleasantness.

#### 3.3.3 Disorganized Schizotypy Is a Specific Predictor of Daily Life Stress‐Reactivity Within Individuals

In the subsequent analysis, we examined whether disorganized schizotypy predicts daily life stress‐reactivity, independent of positive and negative schizotypal traits. We expanded the previous model by incorporating disorganized schizotypy and its interaction with event‐unpleasantness. The analysis revealed that higher levels of disorganized schizotypal traits were linked to elevated event‐unpleasantness and stress‐reactivity (as indicated by the interaction with stressor exposure). Positive schizotypy did not show significant associations with daily life event‐unpleasantness and stress‐reactivity. The significance of negative schizotypy diminished after accounting for disorganized schizotypy. Study days', beep's, age's as well as stressor exposure's main effect on event‐unpleasantness remained significant. For a detailed analysis of the moderation effects of schizotypal traits on stress‐reactivity and the distribution of bootstrapped interaction coefficients, see Table 3 and Figure 3 (second column: B, D, F, H panels).

Moreover, we also examined the relative strength of the effects of disorganized vs. positive as well as disorganized vs. negative schizotypal traits on event‐unpleasantness and stress‐reactivity by analyzing bootstrapped distributions of differences of the coefficients (Figure 4). The results show that, compared to the negative factor, disorganization had a stronger positive effect on event‐unpleasantness in 73% of the cases. Furthermore, compared to the positive factor, disorganization was found to be a stronger positive predictor of event‐unpleasantness in 99% of the differences in bootstrapped coefficients. Regarding stress‐reactivity, disorganization appeared to be a predictor with a larger positive effect nearly 100% of the time compared to negative schizotypy. Finally, in 92% of cases, disorganization was a stronger positive predictor of stress‐reactivity than positive schizotypy. The distribution of coefficient differences is also displayed in detail in Figure 4.

## 4 Discussion

This ESM study investigated how schizotypal traits predict PLE‐ and stress‐reactivity in everyday life. Confirming our expectations, disorganized schizotypy uniquely predicted the increases in both psychotic‐like experiences and the unpleasantness of everyday events in response to elevated stressor exposure within the same three‐day measurement windows. Specifically, participants with higher disorganization showed increased reactivity to stressors in their everyday lifes, which effects were observed over and above positive and negative schizotypy. This finding complements the growing body of literature implicating that disorganization is likely to be a core feature of the schizophrenia‐risk phenotype that may put individuals at enhanced risk to develop psychotic symptoms.

We first established that greater‐than‐usual exposure to social, economic, and health stressors predicted more intense PLEs within individuals. This is in line with previous studies (Evermann et al. 2021; Klippel et al. 2022; Taylor et al. 2019; Wilson et al. 2020) showing that distress is positively associated with psychosis vulnerability. The association further underlines the role of the hypothalamic–pituitary–adrenal (HPA) axis in triggering and exacerbating psychotic symptoms in response to distress (for review, see Pruessner et al. 2017): the HPA is responsible for the production and regulation of cortisol, and beyond elevated overall cortisol levels, cortisol reactivity was also increased in individuals with high genetic risk for psychosis in response to unpleasant events, as compared to healthy controls (Collip et al. 2011).

Next, we tested the hypothesis that disorganized schizotypy predicts PLE‐reactivity in daily life over and above the effects of positive and negative schizotypy (Barrantes‐Vidal et al. 2013; Kemp et al. 2023; Kwapil et al. 2020). Confirming our expectations, while not accounting for disorganized schizotypy, higher positive schizotypy predicted higher PLEs. When disorganized schizotypy was added to the model, positive schizotypy's effect did not remain significant, and disorganized schizotypy predicted higher PLEs and PLE‐reactivity within individuals. Across bootstrap samples, the effect of disorganization was consistently larger than those of positive and negative schizotypy. Furthermore, including disorganization in the model resulted in a significant interaction between negative schizotypy and stressor exposure. In other words, if we hold disorganization constant, individuals with higher negative schizotypy experience lower stress‐reactivity in daily life. This suggests that variance in negative schizotypy not shared with disorganization might be related to blunted psychological responses to stressors, again hinting at the importance of considering disorganization.

Furthermore, our results showed that under greater stressor exposure than usual, individuals appraise events as more unpleasant; thereby, we established stress‐reactivity in the sample. Moreover, higher negative schizotypy was also associated with higher event‐unpleasantness. Importantly, this relationship was, again, moderated by schizotypal traits: elevated positive schizotypy predicted stronger stress‐reactivity; that is, individuals with higher positive schizotypy appraised everyday life events as more unpleasant if they were exposed to stressors. However, upon entering disorganization into the model, it was the sole predictor that moderated stress‐reactivity: event‐unpleasantness showed a stronger association with stressor exposure in more disorganized individuals. Similar to the previous models, across all of the bootstrap samples, disorganization consistently showed stronger positive effects in predicting event‐unpleasantness and stress‐reactivity, as compared to positive and negative schizotypy. This implies that individuals with higher levels of disorganized schizotypy tend to appraise stressful events as more upsetting in daily life within a time window of a few days, while other schizotypal traits did not predict stress‐reactivity after we had accounted for disorganized schizotypy. If the models proposed in this study are valid, these results support our hypothesis that a more negative event appraisal is a consequence of behavioral sensitization, rather than a predictor of it. This may be critical for operationalizing stress‐ and PLE‐reactivity in future studies.

Contrary to our prior expectations, our models revealed no significant independent effect of positive schizotypy either on PLEs or on PLE‐reactivity (although it did predict mean PLE intensity when disorganization was not considered). It should be noted that while we managed to recruit quite a few individuals on the higher ends of the negative and disorganized schizotypy dimensions, the higher range of the positive schizotypy subscale might have been undersampled. This may have prevented us from detecting independent effects of positive schizotypy, and future studies should recruit samples that are enriched in all dimensions of schizotypy.

### 4.1 On the Importance of Disorganization

These results are connected to recent trends in the literature situating disorganization in the context of psychosis risk and general mental health. Previously, in a study that did not assess disorganization, it has been shown that positive and negative schizotypy predicted psychotic reactivity in daily life (Barrantes‐Vidal et al. 2013). However, more and more studies are demonstrating that when disorganization is accounted for, it is uniquely associated with diverse impairments of affective functioning that, if disorganization is left out of the picture, may appear to be related to the positive dimension (Kemp et al. 2023; Kwapil et al. 2020). Our study contributes to this growing body of work. Historically, disorganization has been central in conceptualizations of the schizophrenia‐risk phenotype, and several theorists have argued that it is more proximal to neurodevelopmental anomalies and genetic risk, compared to psychotic features and related subclinical phenomena (Bleuler 1950; Cornblatt and Keilp 1994; Meehl 1989). Relatedly, some research even suggests that disorganization might be linked to a broader, general risk for any kind of psychopathology (i.e., the p‐factor) (Caspi and Moffitt 2018). Therefore, understanding the daily dynamics associated with disorganized schizotypy might be even more broadly relevant for research into individual differences in mental health (Kotov et al. 2020).

Furthermore, these findings—that disorganized schizotypy better explains stress‐reactivity than positive schizotypy—align with studies linking disorganized schizotypy to negative affect (Kemp et al. 2018; Kwapil et al. 2020), neuroticism (Kwapil, Gross, Burgin, et al. 2018), and depressive symptoms (Kemp et al. 2022). This suggests that disorganized schizotypy is associated not only with disturbances in thought, behavior, and communication but also with emotionality and emotion regulation, despite the fact that the MSS‐B disorganized subscale does not include items on emotional dysregulation or affective functioning^2^.

### 4.2 Potential Underlying Mechanisms

Our findings established that disorganization specifically predicts PLEs in response to increased exposure to real‐life stressors. However, it remains an open question whether disorganization causally influences psychotic reactivity or there is a common underlying cause. Relatedly, one may speculate about the mechanisms mediating the association. One possibility might be that disorganization captures the phenomenological aspects of inefficient top‐down control by the prefrontal cortex (Thomas et al. 2021) and/or incoherent cognitive maps in the hippocampus (Musa et al. 2022). In individuals showing increased disorganization and related behavioral signs (i.e., impaired eye movement control), dopamine release in the striatum is less regulated (Soliman et al. 2008; Woodward et al. 2011), especially under stress, and it may cause psychotic experiences.

It is also possible that chronic stressor exposure causes both increased sensitivity to stress and impairments in higher order brain regions that implement control (for review, see Pruessner et al. 2017). According to animal studies, chronic stressor exposure impacts the hypothalamic–pituitary–adrenal axis, leading to structural alterations in key brain regions regulating stress responses (i.e., hippocampus and medial prefrontal cortex) (Cerqueira et al. 2008; Herman et al. 2005; Sapolsky 2000). Chronic distress and high glucocorticoid levels may damage hippocampal cells, contributing to memory and executive function deficits in psychosis, which might be connected to disorganization (Aas et al. 2011; Walder et al. 2000). On a related note, a recent systematic review concluded that a history of childhood trauma was consistently related to higher psychotic reactivity (Bogudzińska et al. 2024), implicating that early and chronic stress exposure might be critical in shaping subsequent sensitivity to stress.

Furthermore, after the inclusion of disorganization, the interaction of negative schizotypy with stressor exposure became significant, indicating that people with higher negative schizotypy experience decreased PLE‐reactivity. A similar effect was also observed in the bootstrapped models predicting stress‐reactivity (negative schizotypy did not have a significant effect on stress‐reactivity in the initial multilevel models). Since negative schizotypy was not a significant predictor of PLE‐reactivity before the inclusion of disorganization, the latter might be a suppressor variable here; however, further theoretical evaluation and statistical analyses are needed in order to interpret the variance in negative schizotypy that is not shared with disorganization.

### 4.3 Limitations and Strengths

Our study also has some limitations. First, it is important to highlight that we did not examine lagged relationships in our study. This was not possible on a momentary basis due to the difference in the measurement frequency of stressors (once every 3 days, retrospective) and PLEs and event‐appraisal (eight times a day, quasimomentary). Therefore, one may argue that the lack of lagged relationships raises questions about our causal model wherein stressors exert a causal effect on PLEs and event‐appraisal. It is possible that someone might be exposed to more stressors due to increased PLEs and negative event‐appraisals, which would imply a reversed direction of causality. This is possible; however, within the context of our current study, some counter‐arguments can be posited: first, it is unlikely that PLEs and negative event‐appraisals would affect stress exposure over such a short, few‐day time window: we believe that psychotic(−like) experiences need to persist longer—i.e., months to years—to cause functional impairment and reduced quality of life (see Dominguez et al. 2011; Rep et al. 2023). In particular, we believe that having elevated PLEs for a couple of days might not be sufficient to cause drastic changes in being able to stay in touch with important others, work‐life balance, or one's financial situation. Second, when our dataset was collected during the pandemic, such stressors could emerge quite suddenly with immediate psychological consequences (see e.g., Simor et al. 2021), supporting our causal assumptions; that is, stressors inducing PLEs and more negative event‐appraisals. However, we fitted lagged models on a 3‐day basis, which showed no significant effects (see Table S7 in Supporting Information for details).

Second, stressor exposure was measured with self‐report, and we did not have the means to verify it objectively (e.g., with financial or housing‐related documents). Moreover, the majority of our final sample consisted of females and, in general, highly qualified individuals, which limits the generalizability of our results.

Some strengths of our study should also be noted: we gathered a diverse population sample representing various age groups, and our study was conducted in a Central‐Eastern European country. This may be of particular importance since psychosis research predominantly focuses on populations from Western societies, exhibiting a notable bias (Burkhard et al. 2021). These factors enhance the generalizability of our findings.

### 4.4 Conclusions

Understanding the impact of contextual factors on the dynamics of neuropsychiatric conditions is crucial since they can influence the emergence and persistence of symptoms. Adopting a dimensional approach, we examined the relationship between schizotypal traits and fluctuations in psychotic‐like experiences and event‐unpleasantness. In order to explore phenomena both at the between‐ and within‐individual levels, we used multilevel models to discover the associations between schizotypal traits, PLEs, unpleasantness reports, and PLE‐ and stress‐reactivity in rich time‐series data collected in the context of everyday life. A possible practical implication of our findings is that they provide further evidence for the predictive validity of the MSS‐B, in that it can identify individuals at increased risk for psychotic‐like experiences; this suggests that the MSS‐B can serve as a screening tool for prodromal assessment (Kwapil et al. 2020). Moreover, these findings contribute to the personalized prediction of shifts in psychological functioning using straightforward self‐reported assessments through digital monitoring of distress and psychosis‐related experiences.

## Author Contributions

**Levente Rónai:** conceptualization, data curation, formal analysis, funding acquisition, investigation, methodology, project administration, resources, software, validation, visualization, writing – original draft, writing – review and editing. **Flóra Hann:** data curation, formal analysis, funding acquisition, investigation, methodology, software, visualization, writing – original draft. **Szabolcs Kéri:** writing – review and editing. **Bertalan Polner:** conceptualization, formal analysis, funding acquisition, methodology, project administration, supervision, validation, writing – original draft, writing – review and editing.

## Conflicts of Interest

The authors declare no conflicts of interest.

## Supporting information


Data S1.

## Endnotes

^1^
Our study has a compliance rate that might appear lower than usual. We argue that the amount of observations we obtained is in fact comparable to literature standards in terms of the absolute number of observations per participant. According to a recent meta‐analysis, a typical ESM design is one with 6 beeps/day for 1 week that can yield a maximum of 42 observations per participant (Wrzus and Neubauer 2023). In our sample, the median number of observations per person was 45, that is, more than the maximum number of observations (42) in typical designs, where 79% is the average compliance (Wrzus and Neubauer 2023). Our study design decisions were constrained by financial limitations, hence our inability to motivate a higher compliance rate in a typical one‐week assessment. In addition, one could argue that our longer study period may enhance the generalizability of the findings, as it may provide a more diverse set of experiences from participants, as compared to the narrower range of experiences that one may report over a week. We further argue that our findings are unlikely to be due to sampling biases, see the Supporting Information (Figure S4 and accompanying text).

^2^
We would like to thank our reviewer Thomas Kwapil for this inspiring remark.
