# Supplementary material for: Thoughts Falling Apart: Disorganized Schizotypy Specifically Predicts Both Psychotic‐ and Stress‐Reactivity in Daily Life
Source: J Pers. 2025 Mar 11;94(1):94–108. doi: 10.1111/jopy.13019 (PMC12780311; doi:10.1111/jopy.13019)
Supplement: Supplementary file 1 — Data S1. [file JOPY-94-94-s001.docx]

Thoughts falling apart: disorganized schizotypy specifically predicts both psychotic- and stress-reactivity in daily life

-

Supplementary Materials

Levente Rónai^1,2^, Flóra Hann^1^, Szabolcs Kéri^3,^, Bertalan Polner^1,4*^

^1^Institute of Psychology, ELTE, Eötvös Loránd University, Budapest, Hungary

^2^Institute of Psychology, University of Szeged, Szeged, Hungary

^3^Sztárai Institute, University of Tokaj, Sárospatak, Hungary

^4^Donders Institute for Brain, Cognition and Behaviour, Radboud University, Nijmegen, The Netherlands

*bertalan.polner@donders.ru.nl

## Participants

### Quality control at the baseline assessment

In order to exclude participants demonstrating low-effort and/or invalid responding, we screened responses in the baseline assessment based on relative completion speed. We calculated the relative speed index according to Leiner (2019), i.e., the median speed of the sample (16 min) divided by the individual’s speed. Then, we excluded observations with a relative speed index greater than 2. As a result, the cutoff speed was set at 8 minutes (med[speed] after screening = 16.24 min).

### Demographic characteristics of participants included vs. with insufficient data

**Table S1**

*Demographic characteristics and comparison of study samples.*

|  | **Initial sample** | **Participants not included** | **Final sample** | **Comparison** |
| --- | --- | --- | --- | --- |
|  |  |  |  | **(not included vs. final)** |
| **Size** | 221 | 117 | 104 |  |
| **Age** |  |  |  | *U* = 5452 |
|  |  |  |  | *p* = 0.183 |
| Mean | 40.44 | 39.3 | 41.68 |  |
| Median | 38 | 37 | 42.5 |  |
| SD | 14.24 | 14.18 | 14.27 |  |
| Range | 18 – 78 | 18 – 78 | 18 – 72 |  |
| **Sex** |  |  |  | *χ²* (1) = 0.059, |
|  |  |  |  | *p* = 0.808 |
| Female % (N) | 73.76 % (163) | 72.65 % (85) | 75 % (78) |  |
| Male % (N) | 26.24 % (58) | 27.35 % (32) | 25 % (26) |  |
| **Education** |  |  |  | *χ²* (5) = 5.083 |
|  |  |  |  | *p* = 0.406 |
| Primary school or lower % (N) | 0.90 % (3) | 0.85 % (1) | 0.96 % (1) |  |
| Vocational school without high school diploma % (N) | 1.81 % (4) | 3.42 % (4) | 0 % (0) |  |
| High school diploma or equivalent % (N) | 23.53 % (52) | 24.79 % (29) | 22.12 % (23) |  |
| Bachelor’s or Master’s degree % (N) | 67.87 % (150) | 64.96 % (76) | 71.15 % (74) |  |
| Doctorate (PhD) % (N) | 4.07 % (9) | 3.42 % (4) | 4.81 % (5) |  |
| Other % (N) | 1.81 % (4) | 2.56 % (3) | 0.96 % (1) |  |

Compliance and schizotypy dimensions

**Table S2**

*Correlations between compliance rates and MSS-B subscales.*

|  | **MSS-B positive schizotypy subscale** | | **MSS-B negative schizotypy subscale** | | **MSS-B disorganized schizotypy subscale** | |
| --- | --- | --- | --- | --- | --- | --- |
|  | Spearman’s rho | *p* | Spearman’s rho | *p* | Spearman’s rho | *p* |
| **Compliance on survey sent every two hours** | -0.071 | .426 | -0.047 | .603 | -0.142 | .114 |
| **Compliance on survey sent every three days** | -0.044 | .623 | -0.076 | .395 | -0.092 | .308 |

## Compliance rates

**Figure S1**

*The distribution of participants’ compliance rates for short two-hour daily surveys during the study.*


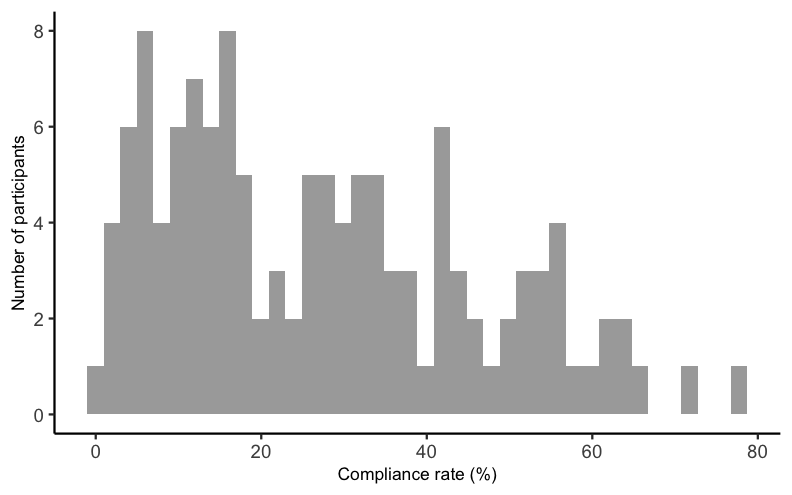


*Note.* Each participant’s compliance rate on the short daily surveys sent every two-hours was calculated against the number of days they spent in the study. For example, if a participant enrolled in the study for 20 days, they received 8 emails for 20 days, totaling 160 surveys for the entire study. So, in this case, the number of completed surveys is divided by 160.

**Figure S2**

*The distribution of participants’ compliance rates for three-day surveys during the study.*


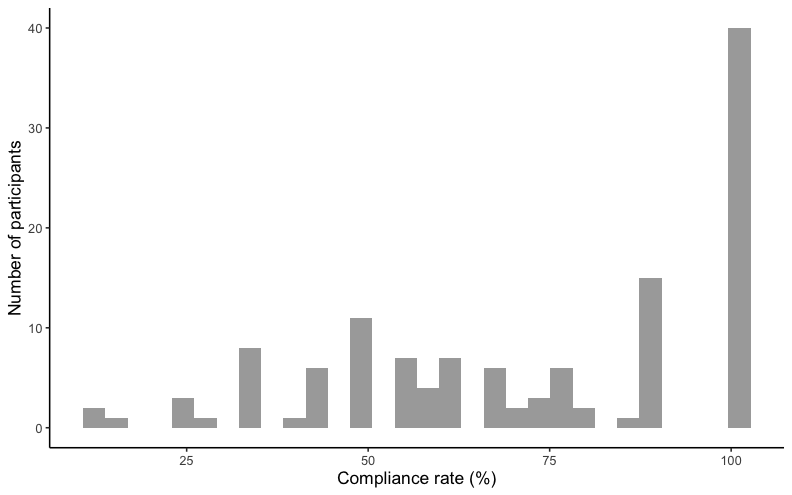


*Note.* Each participant’s compliance rate on the three-day surveys was calculated against the total number of three-day surveys they received during their participation. For example, if a participant enrolled in the study for 20 days, they received 6 three-day surveys in total. So, in this case, the number of completed surveys is divided by 6.

## Instruments and design

### Extracting latent variables for schizotypy with confirmatory factor analysis

**Table S3**

*Factor loadings of MSS-B items based on confirmatory factor analysis.*

| **MSS-B item** | | **Positive schizotypy** | **Negative schizotypy** | **Disorganized schizotypy** |
| --- | --- | --- | --- | --- |
| *35* | *I often worry that someone or something is controlling my behavior.* | 0.802 |  |  |
| *26* | *I often worry that other people are out to get me.* | 0.746 |  |  |
| *32* | *I believe that there are secret signs in the world if you just know how to look for them.* | 0.689 |  |  |
| *14* | *I sometimes wonder if there is a small group of people who can control everyone else's behavior.* | 0.661 |  |  |
| *17* | *I have had the momentary feeling that someone's place has been taken by a look-alike.* | 0.601 |  |  |
| *20* | *There are times when it feels like someone is touching me when no one is actually there.* | 0.520 |  |  |
| *23* | *I have had experiences with seeing the future, ESP or a sixth sense.* | 0.467 |  |  |
| *11* | *I believe that dreams have magical properties.* | 0.451 |  |  |
| *29* | *Some people can make me aware of them just by thinking about me.* | 0.432 |  |  |
| *8* | *I have felt that there were messages for me in the way things were arranged, like furniture in a room.* | 0.353 |  |  |
| *38* | *At times I have wondered if my body was really my own.* | 0.180 |  |  |
| *2* | *I have sometimes felt that strangers were reading my mind.* | 0.175 |  |  |
| *28* | *There are just not many things that I have ever really enjoyed doing.* |  | 0.922 |  |
| *13* | *Throughout my life, very few things have been exciting or interesting to me.* |  | 0.860 |  |
| *16* | *Having close friends is not as important as people say.* |  | 0.740 |  |
| *31* | *I generally am not interested in being emotionally close with others.* |  | 0.740 |  |
| *34* | *My emotions have almost always seemed flat regardless of what is going on around me.* |  | 0.707 |  |
| *4** | *In general, it is important for me to have close relationships with other people.* |  | 0.693 |  |
| *7* | *I have always preferred to be disconnected from the world.* |  | 0.670 |  |
| *37** | *Spending time with close friends and family is important to me.* |  | 0.620 |  |
| *19* | *Generally I do not have many thoughts or emotions.* |  | 0.508 |  |
| *22* | *Throughout my life, I have had little interest in dating or being in a romantic relationship.* |  | 0.488 |  |
| *1* | *Throughout my life I have noticed that I rarely feel strong positive or negative emotions.* |  | 0.472 |  |
| *25** | *Most of the time I feel a desire to be connected with other people.* |  | 0.399 |  |
| *21* | *No matter how hard I try, I can't organize my thoughts.* |  |  | 0.953 |
| *15* | *My thoughts are so hazy and unclear that I wish that I could just reach up and put them into place.* |  |  | 0.906 |
| *24* | *I find that I am very often confused about what is going on around me.* |  |  | 0.906 |
| *36* | *I have trouble following conversations with others.* |  |  | 0.896 |
| *12* | *I often feel so mixed up that I have difficulty functioning.* |  |  | 0.895 |
| *30* | *My thoughts are almost always hard to follow.* |  |  | 0.872 |
| *27* | *People find my conversations to be confusing or hard to follow.* |  |  | 0.864 |
| *6* | *Most of the time I find it is very difficult to get my thoughts in order.* |  |  | 0.839 |
| *18* | *My thoughts and behaviors feel random and unfocused.* |  |  | 0.839 |
| *9* | *I often have difficulty following what someone is saying to me.* |  |  | 0.801 |
| *33* | *I often have difficulty organizing what I am supposed to be doing.* |  |  | 0.705 |

**reversed items*

**Table S4**

*Correlations between MSS-B schizotypy dimensions.*

|  | **Positive schizotypy** | **Negative schizotypy** | **Disorganized schizotypy** |
| --- | --- | --- | --- |
| **Positive schizotypy** | – | 0.125 | 0.544*** |
| **Negative schizotypy** | 0.125 | – | 0.340*** |
| **Disorganized schizotypy** | 0.544*** | 0.340*** | – |

**p<0.05 **p<0.01***p<0.001*

###

### Extracting a between- and within-person latent variable for psychotic-like experiences with multilevel confirmatory factor analysis

**Table S5**

*Multilevel factor loadings of psychotic-like experiences based on multilevel confirmatory factor analysis.*

| **ESM survey item** | **Between-person factor loading** | **Within-person factor loading** |
| --- | --- | --- |
| *I feel like I am losing control.* | 0.769 | 0.740 |
| *I have difficulty controlling my thoughts.* | 0.713 | 0.684 |
| *My thoughts are strange or unusual.* | 0.896 | 0.573 |
| *I am suspicious.* | 0.811 | 0.560 |
| *I feel like I have been mistreated.* | 0.639 | 0.460 |
| *My sight or hearing seems strange or unusual.* | 0.799 | 0.274 |
| *I feel like someone or something is controlling my thoughts or actions.* | 0.637 | 0.453 |
| *Familiar things seem strange or unusual.* | 0.804 | 0.413 |

### Measurement of stressor exposure

Fifteen items described situations to be evaluated on a 5-point Likert scale, 1 meaning that the situation in question significantly worsened in the past 3 days, while 5 meant that the situation improved significantly. Scores of 1–5 were transformed to -2–2, ranging from high support (-2) to high exposure (2). Three items had ‘yes’/’no’ answers, support scoring -2 and exposure 2. The last two items were open questions where participants could describe a source of distress/support that was not mentioned in the survey. Answers to these were manually screened since some descriptions were redundant and could be sorted into the categories of the structured part of the survey. To avoid duplication, these were scored 0. Other sources of support/stress were given scores of -2 and 2, respectively. Finally, scores were summarized, resulting in higher scores meaning higher stressor exposure.

#### Items in Hungarian and English:

Kérjük jelölje be, ha az elmúlt három napban az alábbi események valamelyike megtörtént Önnel! Kérjük, csak azokat jelölje be, amelyek az elmúlt 3 napban történtek meg! Ha valamelyik kérdés nem vonatkozik Önre, nem tudja eldönteni, vagy nem szeretne válaszolni, akkor azt hagyja figyelmen kívül.

English: Please indicate if the following events happened to you in the past three days. Please only mark those that occurred in the last 3 days. If a question does not apply to you, you cannot or do not want to answer, please, ignore that.

*Elvesztettem a munkámat / bevételeim eddigi forrását.*

*I lost my job / source of income.*

*Új munkát / bevételi forrást szereztem.*

*I got a new job / source of income.*

*Jövedelmem: / My income:*

1. jelentős mértékben csökkent. / significantly decreased.
2. valamennyire csökkent. / slightly decreased.
3. nem változott. / did not change.
4. valamennyire növekedett. / slightly increased.
5. jelentősen növekedett. / significantly increased.

*Számomra fontos személy(ek) egészségi állapota a megszokotthoz képest / The health condition of someone/people important to me, as compared to usual,*

1. jelentősen romlott. / significantly worsened.
2. romlott. / worsened.
3. nem változott. / did not change.
4. javult. / improved.
5. jelentősen javult. / significantly improved.

*Saját egészségi állapotom a megszokotthoz képest / My health condition, as compared to usual,:*

1. jelentősen romlott. / significantly worsened.
2. romlott. / worsened.
3. nem változott. / did not change.
4. javult. / improved.
5. jelentősen javult. / significantly improved.

*Számomra fontos személyekkel való kapcsolatom (munkahelyen kívül) a megszokotthoz képest / My relationship with people who are important to me (outside of work), as compared to usual,:*

1. jelentősen romlott. / significantly worsened.
2. romlott. / worsened.
3. nem változott. / did not change.
4. javult. / improved.
5. jelentősen javult. / significantly improved.

*Számomra fontos személyekkel való kapcsolatom a munkahelyen a megszokotthoz képest / My relationship with people who are important to at work, as compared to usual,:*

1. jelentősen romlott. / significantly worsened.
2. romlott. / worsened.
3. nem változott. / did not change.
4. javult. / improved.
5. jelentősen javult. / significantly improved.

*Lakhatási körülményeim / My housing conditions:*

1. jelentősen romlott. / significantly worsened.
2. romlott. / worsened.
3. nem változott. / did not change.
4. javult. / improved.
5. jelentősen javult. / significantly improved.

*Munkahelyemmel / tanulmányaimmal / egyéb kötelezettségeimmel együtt járó terhelés / The burden associated with my job / studies / other obligations:*

1. elentős mértékben csökkent. / significantly decreased.
2. valamennyire csökkent. / slightly decreased.
3. nem változott / did not change.
4. valamennyire növekedett. / slightly increased.
5. jelentősen növekedett. / significantly increased.

*Pihenéssel töltött időm: / The time I spent with rest and relaxation:*

1. jelentős mértékben csökkent. / significantly decreased.
2. valamennyire csökkent. / slightly decreased.
3. nem változott / did not change.
4. valamennyire növekedett. / slightly increased.
5. jelentősen növekedett. / significantly increased.

*Önmagamra fordított idő, amikor bármit csinálhatok, ami örömet okoz, feltölt, vagy a fejlődésemet szolgálja: / The time that I could dedicate to myself, doing anything that brings joy, rejuvenates, or serves my personal development:*

1. jelentős mértékben csökkent. / significantly decreased.
2. valamennyire csökkent. / slightly decreased.
3. nem változott / did not change.
4. valamennyire növekedett. / slightly increased.
5. jelentősen növekedett. / significantly increased.

*Együtt töltött idő számomra fontos személyekkel (olyanokkal, akikkel nem élek együtt) / The time spent with people who are important to me (who I do not live with):*

1. jelentős mértékben csökkent. / significantly decreased.
2. valamennyire csökkent. / slightly decreased.
3. nem változott / did not change.
4. valamennyire növekedett. / slightly increased.
5. jelentősen növekedett. / significantly increased.

*Kapcsolattartás mértéke számomra fontos személyekkel (olyanokkal, akikkel nem élek együtt, pl. telefonon, online, stb.): / The extent of communication with people who are important to me (who I do not live with, and I stay in touch e.g., over the phone, online, etc.):*

1. jelentős mértékben csökkent. / significantly decreased.
2. valamennyire csökkent. / slightly decreased.
3. nem változott / did not change.
4. valamennyire növekedett. / slightly increased.
5. jelentősen növekedett. / significantly increased.

*Anyagi támogatás (családtól, barátoktól, állami támogatás) a megszokotthoz képest: / Financial support (from family, friends, government assistance), as compared to usual:*

1. jelentős mértékben csökkent. / significantly decreased.
2. valamennyire csökkent. / slightly decreased.
3. nem változott / did not change.
4. valamennyire növekedett. / slightly increased.
5. jelentősen növekedett. / significantly increased.

*Munkáimmal / tanulmányaimmal kapcsolatos teendőkkel / egyéb fontos feladataimmal, összességében, mindent egybevéve: / With the tasks related to my work / studies / other important responsibilities, overall, all things considered:*

1. jelentősen lemaradtam. / I fell significantly behind.
2. valamennyire lemaradtam. / I fell slightly behind.
3. nincs változás. / There has been no change.
4. valamennyire előreléptem (pl. kisebb munka befejezése). / I made some progress (e.g., completion of a small task).
5. jelentősen előreléptem (pl. fontos munka befejezése, mérföldkő). / I made significant progress (e.g., completion of an important task, milestone).

*Terveim a tanulással / munkával / egyéb számomra fontos elvégzendő feladattal kapcsolatban: / My plans regarding my studies / work / other important tasks to be completed:*

1. jelentős mértékben felborultak / were significantly disrupted.
2. részben felborultak / were partially disrupted.
3. nem változtak / have not really changed.
4. valamivel átláthatóbbak lettek / became somewhat clearer.
5. jelentősen átláthatóbbak lettek / became significantly clearer.

*A számomra vagy a családom számára szükséges dolgok közül (pl. élelmiszer, számlák, lakbér, gyógyszerek, útiköltség, üzemanyag vagy egy váratlan kiadás: pl. javítatni kellett az autót vagy szerelőt kellett hívni) a megszokotthoz képest: / Among the things necessary for me or my family (e.g., food, bills, rent, medication, travel expenses, unexpected expenses: e.g., having the car repaired, calling a plumber), as compared to usual:*

1. semmit nem tudtam kifizetni / I could not afford anything.
2. egy részét nem tudtam kifizetni / I could not afford some of the necessary things.
3. a szükséges dolgokat ki tudtam fizetni / I could afford the necessary things.
4. a szükségesnél kicsit többet is ki tudtam fizetni / I could afford slightly more than necessary.
5. a szükségesnél jóval többet ki tudtam fizetni / I could afford significantly more than necessary.

*Lekéstem egy fontos találkozóról, eseményről (pl. tárgyalás, orvos). / I was late from an important meeting or event (e.g., negotiation, doctor's appointment).*

*Egyéb jelentős pozitív esemény, változás / Other significant positive event, change:*

*Kérjük, írja le röviden, mi volt az: / Please describe briefly what it was:*

*Egyéb jelentős negatív esemény, változás./ Other significant negative event, change:*

*Kérjük, írja le röviden, mi volt az: / Please describe briefly what it was:*

## Schizotypy scores of participants included vs. with insufficient data

**Table S6**

*Comparison of MSS-B subscale scores between observations included and not included in the final sample.*

|  | **Initial sample** | **Observations not included** | **Final sample** | **Comparison** |
| --- | --- | --- | --- | --- |
|  |  |  |  | **(not included vs. final)** |
| **Size** | 221 | 117 | 104 |  |
| **Disorganized** |  |  |  | *U* = 5906 |
|  |  |  |  | *p* = 0.679 |
| Mean | 1.63 | 1.63 | 1.59 |  |
| Median | 0 | 0 | 0 |  |
| SD | 2.80 | 2.95 | 2.61 |  |
| Range | 0 – 12 | 0 – 12 | 0 – 11 |  |
| **Positive** |  |  |  | *U* = 6183 |
|  |  |  |  | *p* = 0.829 |
| Mean | 1.59 | 1.61 | 1.54 |  |
| Median | 1 | 1 | 1 |  |
| SD | 1.87 | 1.95 | 1.78 |  |
| Range | 0 – 10 | 0 – 10 | 0 – 6 |  |
| **Negative** |  |  |  | *U* = 5683.5 |
|  |  |  |  | *p* = 0.342 |
| Mean | 2.62 | 2.47 | 2.76 |  |
| Median | 2 | 2 | 2 |  |
| SD | 2.43 | 2.39 | 2.48 |  |
| Range | 0 – 12 | 0 – 12 | 0 – 11 |  |

**Figure S3**

*The distribution of PLE factor scores in the sample.*

**
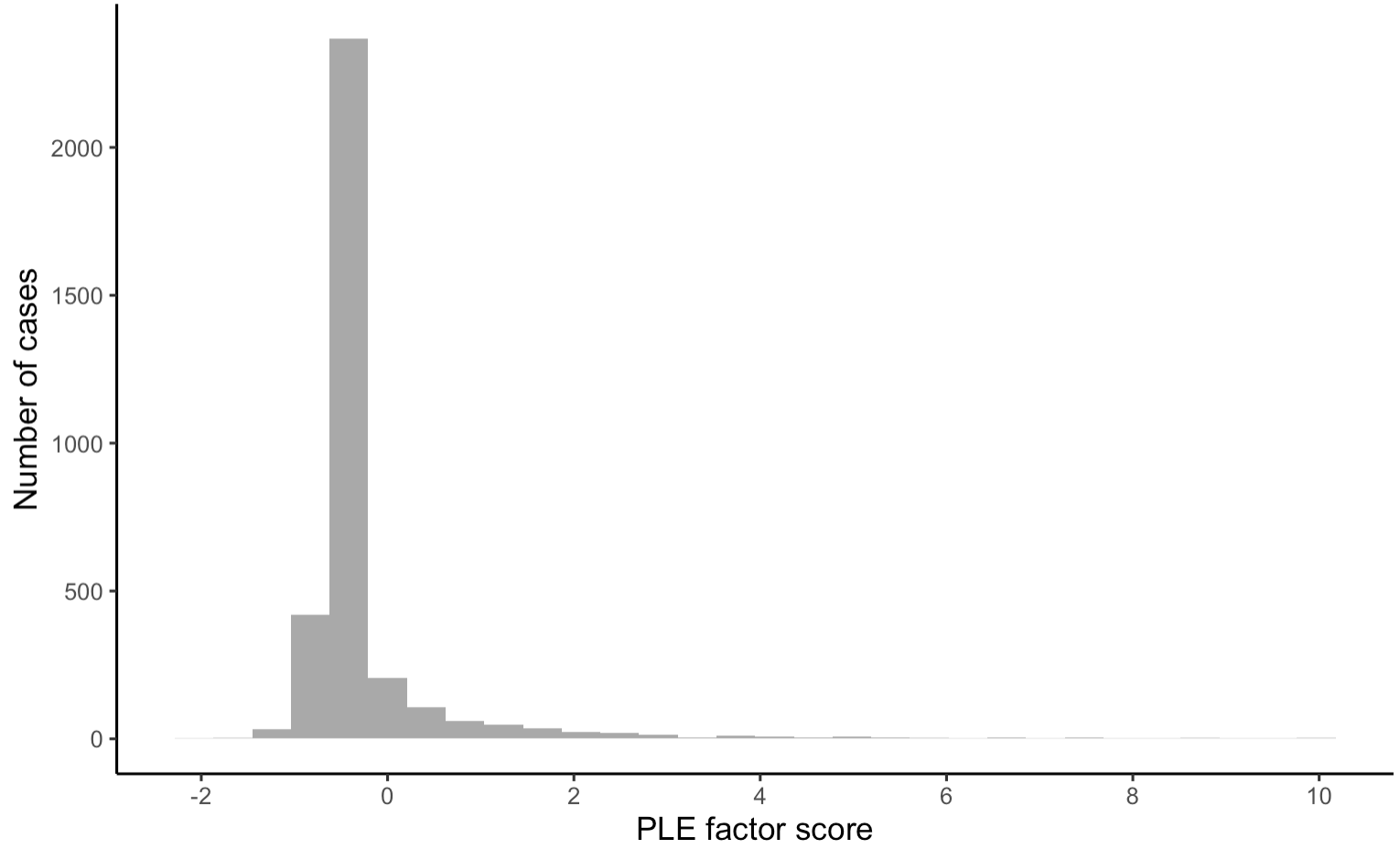
**

*Note.* Each case of PLE factor score corresponds to a single data point (i.e., an ESM survey). Factor scores are based on a multilevel factor analysis of the reported intensity of momentary PLEs.

## Compliance and sampling bias

A lower compliance rate can cause two separate issues that may undermine one’s conclusions. First, it may increase the risk of sampling bias both at the between- and within-participant levels and induce spurious associations. Second, it may reduce the precision of parameter estimation and statistical power due to a lower number of total observations. Here, we provide our evaluation of the potential consequences of our study having a compliance rate that might appear lower than usual.

Regarding between-participant sampling bias, we found no significant correlation between schizotypy scores and compliance rates (also see our response to point 8), which does not raise the concern that our findings could be spurious because of highly schizotypal individuals responding to surveys at a different rate.

Although we cannot rule out a within-participant sampling bias (e.g. everyone selectively completes ESM surveys as a function of current stress or PLE), we can reflect on how likely it is that the key study findings are induced by such a bias. For example, PLE-reactivity to stressor-exposure could emerge as an artefact if there were no true relationship but participants selectively responded only at times when both PLEs and stressor-exposure were both either low or high, but skip ESM surveys when only one of them was high but the other one was low (e.g. high stressor exposure and low PLE, and vice versa). We believe that such a selective responding pattern is unlikely.

What we find more possible is that due to their distracting nature, elevated PLEs and stressor-exposure could both decrease the likelihood of completing an ESM survey, and this might be more pronounced for disorganized individuals. Such a tendency would actually induce a negative spurious association between stressor-exposure and PLEs (i.e., a collider bias). This would attenuate the estimated association between stressor exposure and PLEs, and the positive effect of disorganization on PLE-reactivity as well. We visualize our reasoning below (see Figure S4): we present a simulated dataset, assuming a true linear positive association between PLEs and stressors, without any within-participant sampling bias (on the left), and with the above-described sampling bias (on the right, the darker the coloring, the less likely an experience to be captured in the dataset). Note how the positive correlation is attenuated if one considers only the dots in the yellow and green areas in the figure on the right.

**Figure S4**

*Demonstration of within-participants sampling bias on simulated data*


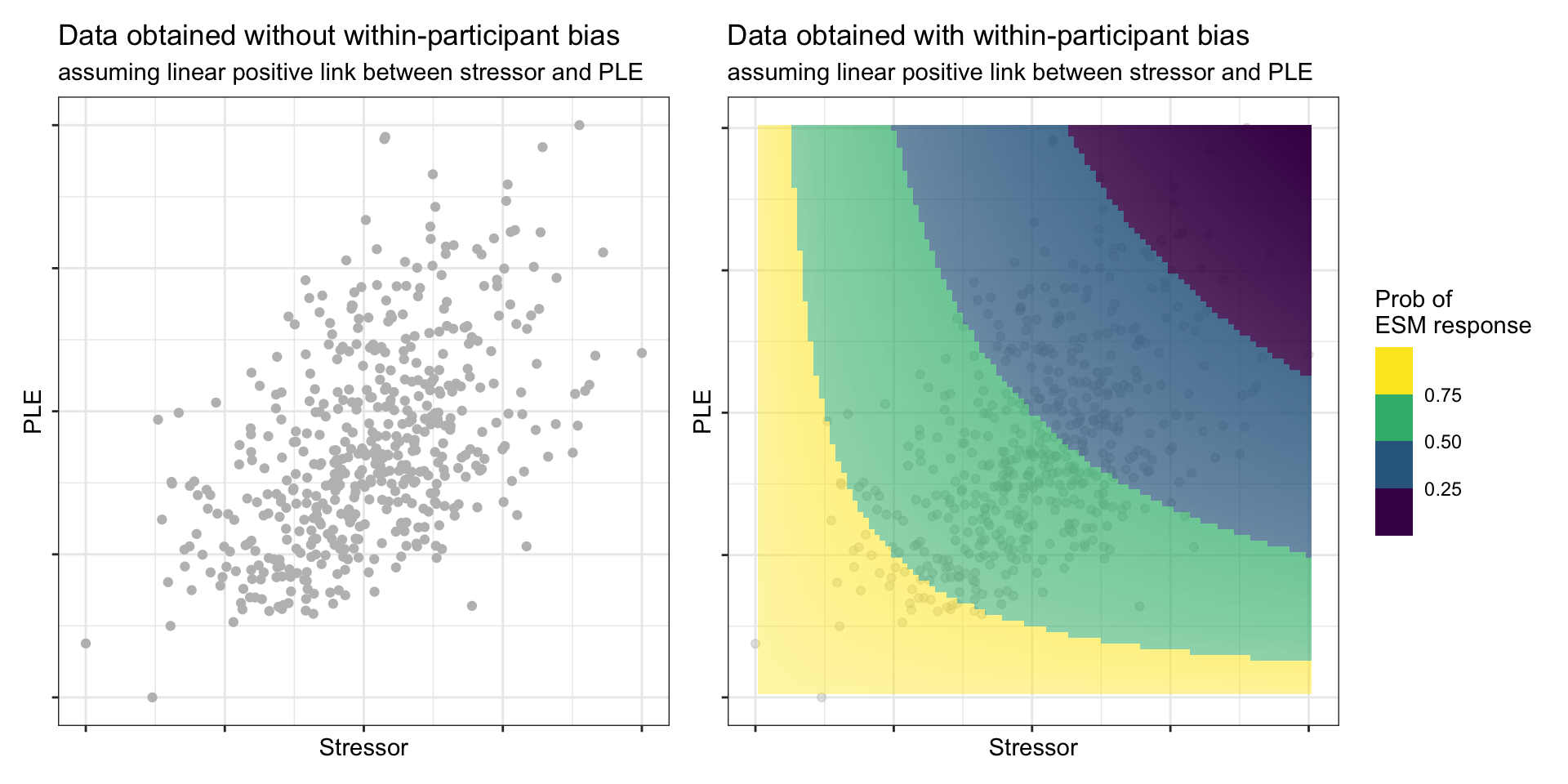


### Causality and lagged relations

To test the possible lagged relationships, we fitted lagged models (Table S7). First, we examined whether stressors assessed in the previous 3-day time window predict PLEs and event unpleasantness in the present 3-day time window. We also examined how PLEs and event unpleasantness measured in the previous 3-day time window predict stressors for the present 3 days (see the table summarizing the results, from left to right). The results show that there are no significant lagged relationships in either direction.

We performed additional analyses to empirically support or discard our causal assumptions. We examined the relationships between event-appraisal, PLE, and stress-exposure by fitting partial correlations for each three-day time window within individuals. Our result indicated that the median correlation coefficient (r) between mean levels (i.e. means within 3-day time windows) of PLEs and event-appraisal was 0.32 within individuals. However, when controlled for stress-exposure as a confounder (i.e., a common cause of PLEs and stress-appraisal), the magnitude of the relationship decreased remarkably (median r = 0.13). To test another combination (i.e., PLE as a confounder), the association between stress-exposure and event-appraisal was investigated. In this case, the median r was 0.21 within individuals, while after adjusting for 3-day-mean levels of PLEs as a common cause, the strength of the correlation remained almost unchanged between stress-exposure and event-appraisal (median r = 0.17). This supports the assumption in our causal model that stress-exposure may affect PLEs and event-appraisal (and their relationship), while PLEs might not affect changes in stress-exposure and event-appraisal (and the relationship between the latter two) in the three-day measurement windows within individuals in our sample. To sum up, in our view it seems justified to model stress-exposure as a predictor and PLEs and event-appraisal as outcome variables in three-day time windows. Still, an inverse relationship between the former variables is also plausible at other temporal resolutions.

**Table S7**

*Lagged (outcome [present 3-day window] ~ predictors [previous 3-day window]) associations of stressor-exposure, PLEs and event-appraisal.*


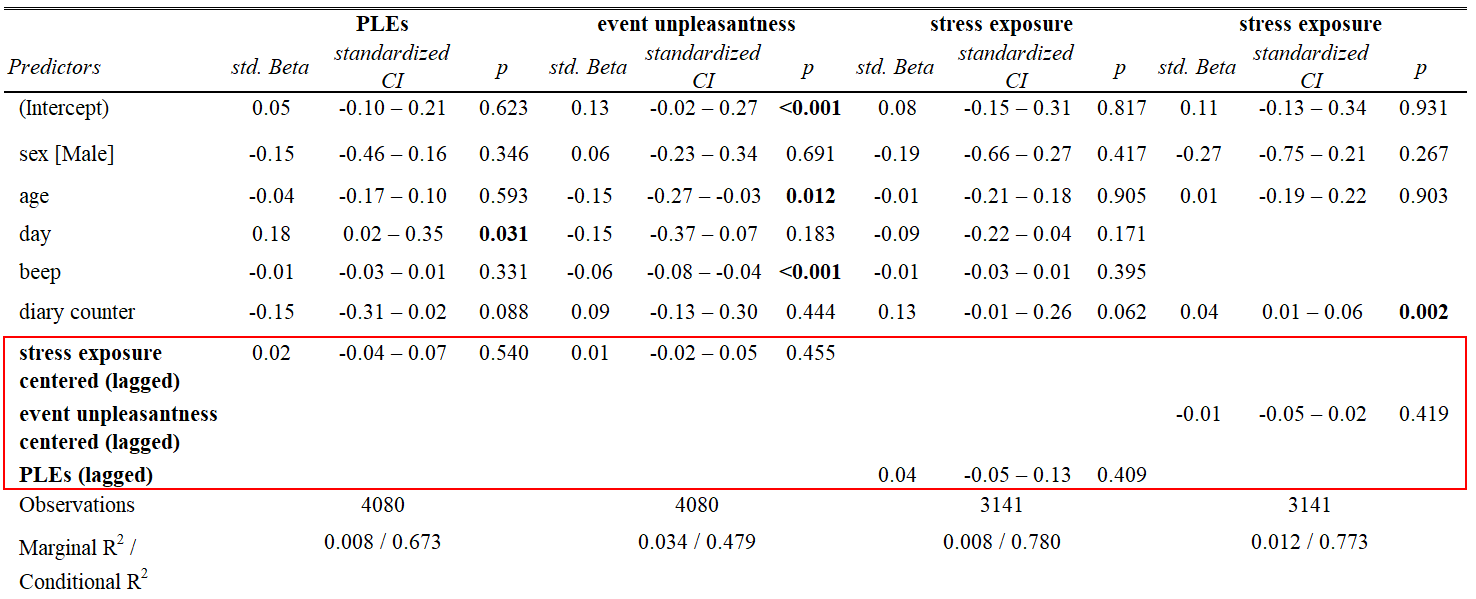


###

# References

Leiner, D. J. (2019). Too Fast, too Straight, too Weird: Non-Reactive Indicators for Meaningless Data in Internet Surveys. *Survey Research Methods*, *13*(3), Article 3. <https://doi.org/10.18148/srm/2019.v13i3.7403>
